# Enhancement of Gene Silencing Effect and Membrane Permeability by Peptide-Conjugated 27-Nucleotide Small Interfering RNA

**DOI:** 10.3390/molecules170911089

**Published:** 2012-09-14

**Authors:** Takanori Kubo, Kazuyoshi Yanagihara, Yuichiro Sato, Yasuhiro Morita, Toshio Seyama

**Affiliations:** 1 Faculty of Pharmacy, Yasuda Women’s University, 6-13-1 Yasuhigashi, Asaminami-ku, Hiroshima 731-0153, Japan; Email: sato-y@yasuda-u.ac.jp (Y.S.); morita-y@yasuda-u.ac.jp (Y.M.); seyama@yasuda-u.ac.jp (T.S.); 2 Division of Genetics, National Cancer Center Research Institute, 5-1-1 Tsukiji, Chuo-ku, Tokyo 104-0045, Japan; Email: kyanagih@ncc.go.jp

**Keywords:** peptide conjugates, Dicer-substrate, 27-nt siRNA, potent gene silencing, intracellular delivery

## Abstract

Two different sizes of siRNAs, of which one type was 21-nucleotide (nt) siRNA containing 2-nt dangling ends and the other type was 27-nt siRNA with blunt ends, were conjugated with a nuclear export signal peptide of HIV-1 Rev at the 5′-sense end. Processing by Dicer enzyme, cell membrane permeability, and RNAi efficiency of the peptide-conjugated siRNAs were examined. Dicer cleaved the peptide-conjugated 27-nt siRNA leading to the release of 21-nt siRNA, whereas the peptide-conjugated 21-nt siRNA was not cleaved. High membrane permeability and cytoplasmic localization was found in the conjugates. Moreover, the peptide-conjugated 27-nt siRNA showed increased potency of RNAi in comparison with the nonmodified 21-nt and 27-nt siRNAs, whereas the peptide-conjugated 21-nt siRNA showed decreased RNAi efficacy. This potent RNAi efficacy is probably owing to acceleration of RISC through recognition by Dicer, as well as to the improvement of cell membrane permeability and intracellular accumulation.

## 1. Introduction

The technology of RNAi has attracted particular attention as the most powerful tool for suppressing gene expression in mammalian cells [1,2]. Fire *et al*. discovered the phenomenon of RNAi using a long double-stranded RNA (dsRNA), which induced the sequence-specific degradation of homologous mRNAs [3]. In the cytoplasm of mammalian cells, a long dsRNA is cleaved to short dsRNAs by a Dicer enzyme [4,5]. Short dsRNAs of 21 to 23 nucleotides (nt) in length, having a phosphate at the 5′-end and a 2-nt overhang at the 3′-end, are named small interfering RNAs (siRNAs). These siRNAs are bound to a protein complex called an RNA-induced silencing complex (RISC) [6]. The RISC cleaves the target mRNA at a sequence-specific position, guided by the antisense strand of the siRNAs. Because RNAi works in a sequence-specific manner, strong gene suppression requires only a few siRNA molecules.

However, in mammalian cells, a long dsRNA induces an interferon response [7]. To prevent interferon activation, Elbashir *et al*. have used chemically synthesized 21-nt siRNAs, which consist of a 19-nt duplex in the central region and a 2-nt overhang at the 3′-end [8]. Since 21-nt siRNAs prevented interferon activation and strongly affected gene expression (similar to the effect of long dsRNA), chemically synthesized 21-nt siRNAs are widely used [9,10,11,12,13].

Although RNAi has many advantages over other genetic drug technologies, several problems, such as cellular delivery, stability against nuclease degradation, and side effects (off-target effects and interferon responses at high concentrations), must be solved before it can be applied in the clinic [14,15,16,17,18,19,20]. To solve the problems associated with RNAi, by improving the properties of 21-nt siRNAs, many chemically modified 21-nt siRNAs have been developed [21,22,23,24,25,26,27]. It has been reported that amino-modified 21-nt siRNAs at the 3′-end are highly stable against nuclease degradation [21]. The 2′-modifications (2′-O-Me and 2′-F) of 21-nt siRNAs and sugar modifications [e.g., locked nucleic acid (LNA)] also demonstrate high nuclease stability [22,23,24]. Modification with unlocked nucleic acids (UNA), which is an acyclic nucleotide splitting the 2′-3′-bound, either at an overhang or at a specific position of the siRNAs, showed enhanced potency of RNAi with high stability and reduced off-target effects [25]. The modifications of a phosphate backbone (e.g., phosphorothioate and boranophosphate) result in high nuclease stability and strong gene suppression [26,27]. Direct conjugation with functional molecules, including polymers [28,29], peptides [30,31,32,33], and lipids [34,35,36,37,38], to 21-nt siRNAs has been reported to improve the biological properties of siRNAs *in vitro* and *in vivo*. Polyethylene glycols (PEGs)-siRNA conjugate showed much greater enhancement of stability against nuclease than nonmodified siRNAs [29]. To facilitate membrane permeability, cell-penetrating peptides (CPPs), such as TAT trans-activator protein from human immunodeficiency virus type-1 (HIV-1) and Penetratin from the homeodomain protein of *Antennapaedia*, were covalently conjugated to siRNAs using a heterobifunctional cross-linker [30,31]. Covalent conjugation of cholesterol, bile acids, and long-chain fatty acids to siRNAs at the 3′-end of the sense strand were also synthesized and mediated siRNA uptake in cell *in vitro* and *in vivo* [34,35,36,37,38]. These lipophilic siRNAs interacted with lipoprotein particles, lipoprotein receptors, and transmembrane proteins, and influenced uptake behaviors of the siRNAs. These conjugated siRNAs exhibited RNAi activity without any transfection reagent, but only at high (micromolar) concentrations. Although the modifications and conjugations of 21-nt siRNAs, as described above, could solve some of the problems of RNAi, such as nuclease stability or cell permeability, most of them weaken the gene-silencing efficacy. 

On the other hand, it was recently found that blunt-ended 27-nt siRNAs have a much stronger gene silencing effect than 21-nt siRNAs [39,40]. The 27-nt siRNAs are cleaved by a Dicer enzyme, leading to the release of 21-nt siRNAs, and thus are incorporated into the RISC. We previously reported that the 27-nt siRNAs modified with amine at the 5′-end of the sense strand exhibited much better RNAi potency compared with the nonmodified 27-nt siRNAs [41,42,43].

In the present study, we synthesized 27-nt siRNA conjugated with nuclear export signal (NES) peptide, which used the human immunodeficiency virus type-1 (HIV-1) Rev sequence that consist of short amino acid sequences rich in leucine and the other aliphatic amino acids [44], at the 5′-end of the sense strand, and investigated its biological properties, such as prossecing by Dicer enzyme, cell membrane permeability, and RNAi efficacy.

## 2. Results and Discussion

### 2.1. Results

#### 2.1.1. Synthesis of 21-nt and 27-nt siRNAs Conjugated with Peptide

**Scheme 1:**
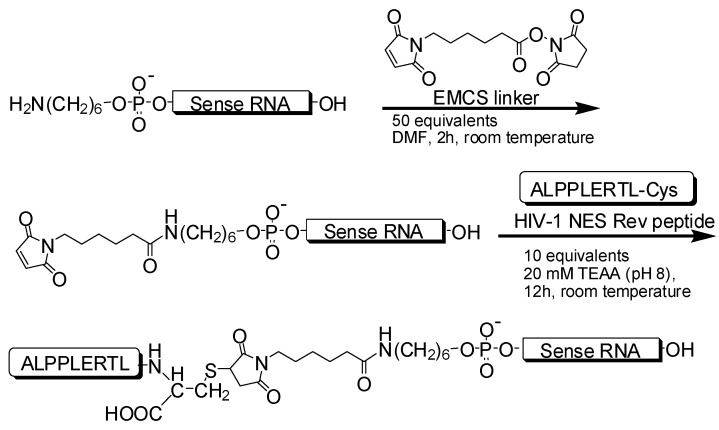
Synthesis of peptide-conjugated ssRNAs.

**Figure 1:**
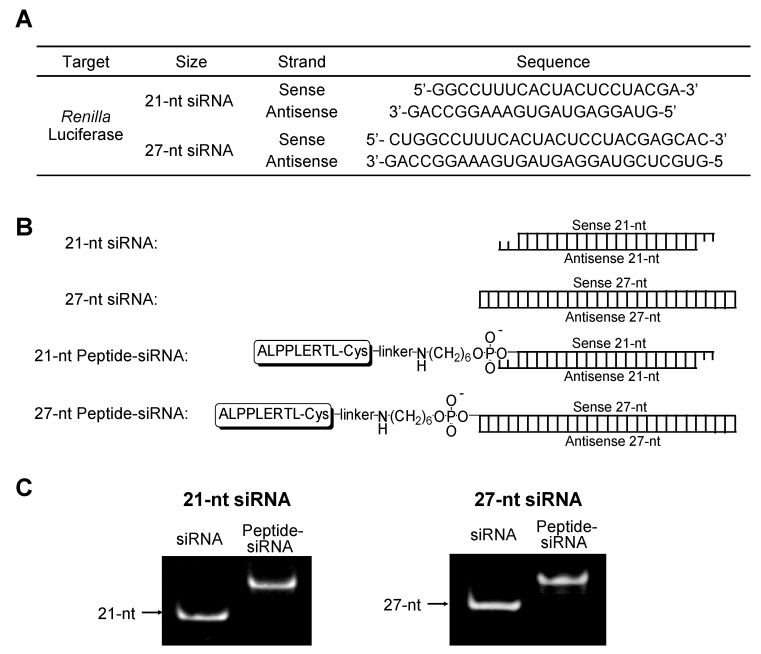
(**A**) Sequences of siRNAs. Two different sizes of siRNAs (21-nt and 27-nt) were designed and targeted to exogenous *Renilla* luciferase; (**B**) Structures of Peptide-siRNAs. HIV-1 Rev NES peptide was covalently attached to the 21-nt and 27-nt siRNAs at the 5′-end of the sense strand; (**C**) PAGE analysis of 21-nt Peptide-siRNA and 27-nt Peptide-siRNA.

Amino-modified 21-nt and 27-nt single-stranded RNAs (ssRNAs) at the 5′-end corresponding to the sense-strand of the target site of *Renilla* luciferase gene were conjugated with HIV-1 Rev NES peptide via an *N*-(6-maleimidocaproyloxy)succinimide (EMCS) linker (Scheme 1). Conjugates were purified by RP-HPLC, and the molecular masses were identified by MALDI-TOF mass spectrometry (Table 1). The peptide-conjugated 21-nt and 27-nt ssRNAs were obtained in 33.0% and 22.9% overall yields, respectively, after HPLC purification (Table 1), and were annealed with their antisense strands of 21-nt and 27-nt ssRNAs, respectively (Figure 1B). The 21-nt and 27-nt peptide-conjugated siRNAs (Peptide-siRNAs) were analyzed by 20% polyacrylamide gel electrophoresis (PAGE), and each corresponding band showed a different mobility in comparison with that of nonmodified 21-nt and 27-nt siRNAs (Figure 1C). These Peptide-siRNAs were clearly separable, and their purity for further use was confirmed.

**Table 1:** Characterizations of ssRNAs conjugated with peptide.

Size	Target gene	Conjugate	HPLC retention time ^a^ (min)	MALDI-TOF MS ^b^ Found/Calcd	Yield ^c^ (%)
21-nt ssRNA	Luciferase	None	6.5	6569.8/6569.9	d
21-nt ssRNA	Luciferase	HIV-1 Rev	15.5	8154.5/8153.8	33.0
27-nt ssRNA	Luciferase	None	6.9	8463.0/8465.1	d
27-nt ssRNA	Luciferase	HIV-1 Rev	16.8	10,057.5/10,049.0	22.9

^a^ A linear gradient condition of acetonitrile shifting the concentrations from 7% to 70% during 40 min in 20 mM triethylammoniumacetate (pH 7.0) using an ODS column; ^b^ A saturated solution of 2,4,6-trihydroxyacetophenone in 50 mg/mL diammonium hydrogen citrate in 50% acetonitrile was used as a matrix; ^c^ Overall yields of the products were determined by measuring absorbance at 260 nm after RP-HPLC purification; ^d^ The purified ssRNAs were purchased.

#### 2.1.2. Processing of Peptide-siRNAs by Dicer

**Figure 2:**
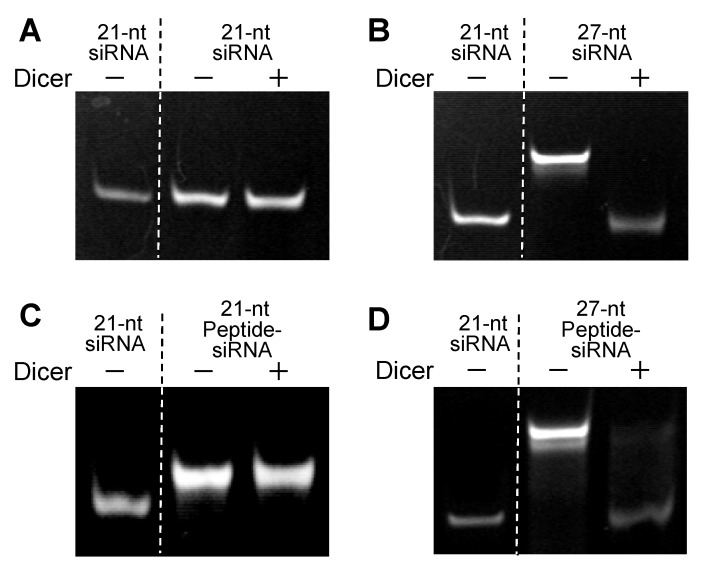
Dicer-substrate siRNAs including peptide conjugates. 21-nt siRNA (**A**), 27-nt siRNA (**B**), 21-nt Peptide-siRNA (**C**), and 27-nt Peptide-siRNA (**D**) were reacted with the recombinant Dicer enzyme for 12 h at 37°C. The reaction products were electrophoresed on 20% PAGE and visualized by silver staining.

The *in vitro* cleavage by Dicer of Peptide-siRNAs to a 21-nt siRNA was investigated. The 27-nt Peptide-siRNA was cleaved to a 21-nt siRNA after Dicer digestion, whereas the 21-nt Peptide-siRNA was intact under the same protocols; that is, no Dicer digestion occurred on the 21-nt Peptide-siRNA (Figure 2). These results suggested that the peptides conjugated to the 5′-end of the 27-nt siRNA did not obstruct Dicer recognition.

#### 2.1.3. Gene Silencing of Peptide-siRNAs

**Figure 3:**
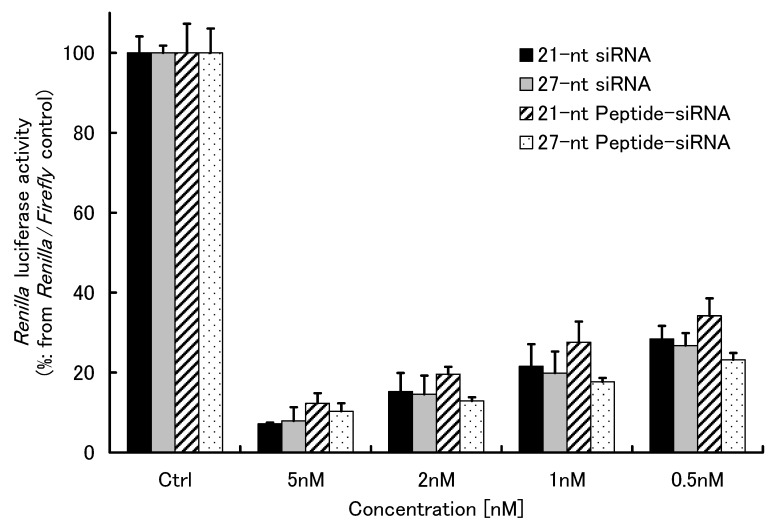
Comparative study of RNAi efficacy of Peptide-siRNAs and nonmodified siRNAs. The Peptide-siRNAs and nonmodified siRNAs (0.5, 1, 2, and 5 nM each) were transfected by LF2000 to HeLa cells, and controls were given only PBS(−). RNAi efficacies of the Peptide-siRNAs and nonmodified siRNAs were evaluated to detect the luminescence of *Renilla* luciferase activity, which was normalized by the luminescence of *Firefly* luciferase activity, after 48 h incubation. The mean and SD values are from three independent experiments.

To evaluate the gene-silencing efficacy of peptide-conjugates, we selected an exogenous *Renilla* luciferase as a target gene, because the luciferase reporter assays were among the conventional approaches for RNAi. We compared the RNAi potency of 21-nt and 27-nt Peptide-siRNAs, including nonmodified siRNAs, targeted to the *Renilla* luciferase gene in the presence of Lipofectamine 2000 (LF2000). *Renilla* luciferase gene expression was dose-dependently suppressed in all samples with high potency (Figure 3). Among them, the 27-nt Peptide-siRNA was the most effective for gene silencing of the exogenous *Renilla* luciferase activity in HeLa cells. However, the 21-nt Peptide-siRNA showed lower RNAi potency compared with nonmodified siRNAs. Thus, modifying 21-nt siRNA with peptide may affect adversely the ability of the molecules to silence genes. These results indicate that the 27-nt Peptide-siRNA is a more attractive molecule than 21-nt Peptide-siRNA for application to RNAi technology. 

#### 2.1.4. Cell Membrane Permeability of Peptide-siRNAs

**Figure 4:**
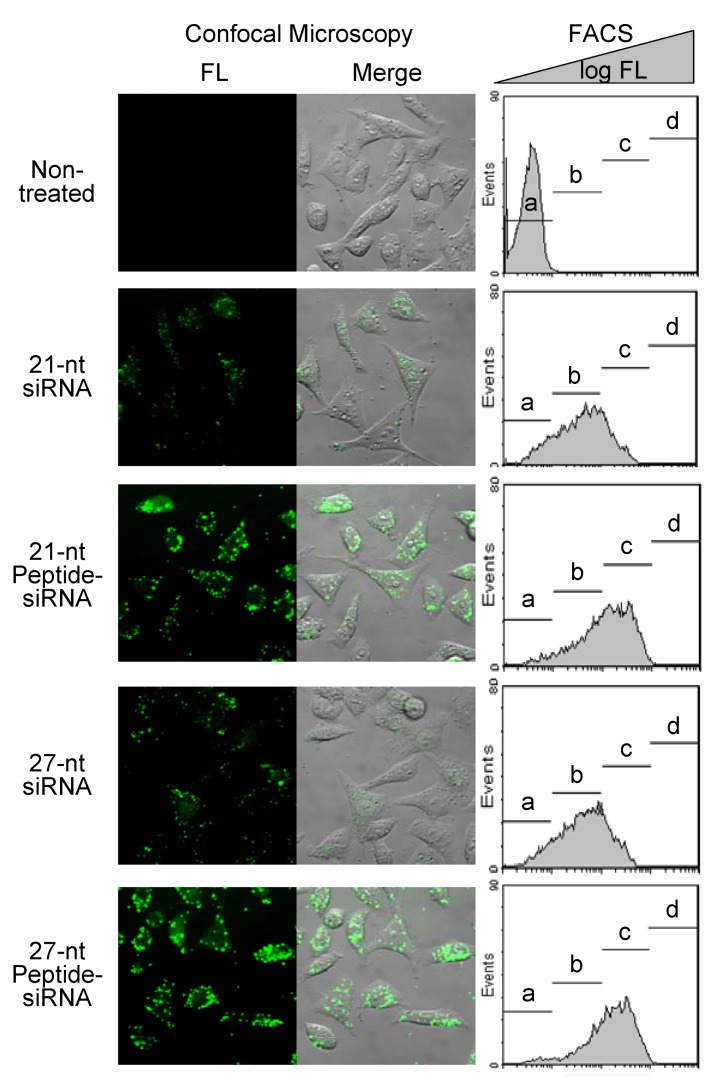
Confocal microscopic images and FACS analysis of HeLa cells incubated for 6 h with 21-nt and 27-nt Peptide-siRNAs (200 nM), including nonmodified siRNAs, labeled with FAM in the presence of LF2000. FL, fluorescence image; Merge, merged image of FL and Transmission; FACS, FACS analysis. In the FACS analysis, the logarithm of the fluorescence intensity is shown on the horizontal axis, and the number of cells is shown on the longitudinal axis. The percentage of cell population separated into four parts (**a**–**d**) along with the fluorescence intensity.

The membrane permeability of 21-nt and 27-nt Peptide-siRNAs, including nonmodified siRNAs, was investigated in HeLa cells in the presence of LF2000 using confocal microscopy and flow cytometry. In the observation by confocal microscopy, the cells treated with siRNAs including peptide conjugates, all of which were labeled with fluorescence (FAM) at the 5′-end of the antisense strand, exhibited bright fluorescence (Figure 4). Among them, both 21-nt Peptide-siRNA and 27-nt Peptide-siRNA labeled with FAM exhibited extremely high fluorescence intensity in the cytoplasm of HeLa cells. The results of flow cytometric analysis showed the same tendency as the microscope observations. In the flow cytometric analysis, the area of the histogram is separated into four populations (**a**–**d**) along with the fluorescence intensity. The percentages of cell populations are shown in Table 2. The histograms of the HeLa cells treated with 21-nt and 27-nt Peptide-siRNAs labeled with FAM exhibited higher fluorescence-intensity populations than the histograms of the cells treated with nonmodified 21-nt and 27-nt siRNAs. Especially, high percentages of cells treated with 27-nt Peptide-siRNA labeled with FAM were distributed at high-fluorescence-intensity populations in the histograms. Accordingly, the Peptide-siRNAs showed enhanced cell membrane permeability and cytoplasmic accumulation in culture cells, as predicted by the properties of the peptide. 

**Table 2:** Fluorescence-intensity populations (**a**–**d**) of the HeLa cells treated with siRNAs by flow cytometric analysis.

siRNAs	Populations of the HeLa cells (%)			
	a	b	c	d
Non-treated	99.58	0.40	0.01	0.00
21-nt siRNA	16.44	60.36	21.89	0.00
21-nt Peptide-siRNA	7.35	36.57	55.00	0.31
27-nt siRNA	14.37	62.29	22.30	0.00
27-nt Peptide-siRNA	5.62	28.93	64.07	0.49

### 2.2. Discussion

Direct conjugations of the peptides to siRNAs are considered to enhance the potency of RNAi, facilitating the cellular penetration of the attached siRNAs. It has been reported that siRNAs conjugated with CPPs, such as TAT trans-activator protein from HIV-1 [30], Penetratin from the homeodomain protein of *Antennapedia* [31], demonstrated enhanced delivery to target cells. Although the CPP-siRNA conjugates have an advantage for intracellular delivery, the RNAi efficacy of the conjugates was reduced in comparison with nonmodified siRNAs using a cationic liposome-based cell-transfection methodology [31]. Our data also showed that 21-nt Peptide-siRNA reduced RNAi efficacy in the presence of LF2000. One of the reasons for the reduced RNAi efficacy is that the peptide might interrupt the incorporation of RISC into the siRNAs. Several papers have been reported that the termini-conjugates or modified 21-nt siRNAs decreased RNAi potency [21,44]. The 21-nt siRNAs conjugated with NH_2_ or iB modifications at all four termini reduced RNAi potency for luciferase activity in the presence of LF2000 [21]. Other modifications, such as 3′-puromycine and 3′-biotin-modified-21-nt siRNA, also reduced RNAi potency for GFP gene expression in the presence of LF2000 [45]. To avoid this, cleavable linkages, such as the disulfide linkage, were used for the conjunction of siRNA and peptide [46]. 

In this study we focused on the blunt-ended 27-nt siRNAs, which were cleaved by Dicer, leading to release of a 21-nt siRNAs, and we developed 27-nt Peptide-siRNA to overcome the problems of siRNAs. We expected the 27-nt Peptide-siRNA to have high membrane permeability, intracellular accumulation, and a Dicer substrate cleaved to the 21-nt siRNA, leading to the release of peptide molecules. Peptide conjugated to siRNA was restricted to the 5’-end of the sense strand based on our previous reports [41,42,43].

The 27-nt Peptide-siRNA appeared to be cleaved to a 21-nt siRNA after reaction with Dicer. We predicted that the 27-nt Peptide-siRNA do not obstruct certain functions of RISC. In contrast, the Dicer did not recognize 21-nt Peptide-siRNA. Thus, 21-nt Peptide-siRNA could adversely affect the mechanism underlying RNAi’s action, which may be ascribed to either their lowered affinity to the RISC complex or their disabling of the complex. In fact, our data showed the 27-nt Peptide-siRNA exhibited a high level of gene silencing (Figure 3), whereas the 21-nt Peptide-siRNA showed slightly disabled ability in comparison with nonmodified siRNA. 

Other factors in the increased RNAi of the 27-nt Peptide-siRNA are high membrane permeability and intracellular accumulation. Although the 21-nt Peptide-siRNA has high cell membrane permeability, the 27-nt Peptide-siRNA appeared to be superior to the 21-nt Peptide-siRNA in cell membrane permeability in HeLa cells in the presence of LF2000. Moreover, the 27-nt Peptide-siRNA, containing NES as a peptide, was accumulated in the cytoplasm. This cytoplasmic accumulation of Peptide-siRNAs is one of the important elements for RNAi technology, because the siRNAs should work in the cytoplasm.

## 3. Experimental

### 3.1. Synthesis of siRNAs Conjugated with NES Peptide

The sequences of siRNAs were designed to target the synthetic *Renilla* luciferase gene (Figure 1A). ssRNAs (21-nt and 27-nt), including 5′-amino-modified ssRNAs, were purchased from Integrated DNA Technologies (Coralville, IA, USA). The amino-modified ssRNAs (sense strand; 4 nmol) were reacted with 200 nmol EMCS linker (Dojindo, Kumamoto, Japan) in *N,N*-dimethylformamide (DMF)/ water mixtures for 2 h at room temperature. The ssRNAs coupled with the EMCS linker were purified using a Poly-Pak cartridge (Glen Research, Sterling, VA, USA) to remove any excess linker, and were used for peptide conjugation. The peptide in the present study was the NES peptide of HIV-1 Rev with a cysteine residue at the C-terminal (ALPPLERLTL-Cys). The coupling reaction of the peptide (40 nmol) and the EMCS-linked ssRNAs (4 nmol) mentioned above were carried out in 20 mM triethylammoniumacetate (TEAA) (pH 8.0) for 12 h at room temperature (Scheme 1). The peptide-ssRNA conjugates were purified by RP-HPLC. The molecular weights of the peptide-conjugated ssRNAs were confirmed by MALDI-TOF mass spectrometry (Ultraflex; Bruker Daltonics, Bremen, Germany) as predicted. The yield of the conjugates was spectrophotometrically calculated on the basis of absorbance at a 260 nm wavelength (V-670 spectrophotometer; JASCO, Tokyo, Japan). Antisense ssRNAs (21-nt and 27-nt) for the *Renilla* luciferase gene were prepared in order to generate Peptide-siRNAs in conjunction with the sense strands of the peptide-conjugated ssRNAs mentioned above.

### 3.2. *In Vitro* Cleavage of Peptide-siRNAs by Recombinant Dicer

All of the siRNAs (20 pmols each) including the peptide conjugates were mixed with 1 U of recombinant Dicer (Gene Therapy Systems, San Diego, CA, USA) in 10 μL of 20 mM Tris-HCl (pH 8.0) containing 15 mM NaCl and 2.5 mM MgCl_2_. The mixtures were incubated at 37 °C for 12 h, and the reaction was stopped by adding 2 μL of stop solution (Gene Therapy Systems). The reaction products were electrophoresed on 20% PAGE (30 mA, 70 min) and visualized by silver staining (DNA Silver Stain Kit; GE Healthcare, Piscataway, NJ, USA). The signals of the reacted products were photographed by the LAS4000 imaging system (Fujifilm, Tokyo, Japan).

### 3.3. Cell Culture

HeLa cells, which were obtained from the RIKEN Bioresource Center Cell Bank (Tsukuba, Japan), were cultured in Dulbecco’s modified Eagle’s medium (DMEM; Wako, Osaka, Japan), supplemented with 10% heat-inactivated FBS (Invitrogen, La Jolla, CA, USA), 100 U/mL penicillin, and 100 μg/mL streptomycin (Wako). The cells were cultured in a humidified atmosphere of 5% CO_2_ in air at 37 °C.

### 3.4. Transfection of Peptide-siRNAs Targeting Renilla Luciferase mRNA

The HeLa cells were adjusted to 1 × 10^5^ cells/mL in 100 μL medium in each well of 96-well multiplates and were cultured. Twelve hours later, 0.02 μg of psiCHECK-2 vector (Promega, Madison, WI, USA) was first incubated with 0.2 μL of LF2000 (Invitrogen) in 10 μL of Opti-MEM (Invitrogen) for 30 min according to the manufacturer’s protocol, and then 10 μL of the mixture was added to each well containing the cells in 90 μL fresh culture medium without antibiotics. To investigate RNAi, the Peptide-siRNAs at different concentrations (0.5, 1, 2 and 5 nM) were pre-incubated with LF2000. Four hours after transfection of the vectors, 10 μL of the pre-incubated mixture of Peptide-siRNAs and LF2000 was added to each well containing 90 μL fresh culture medium. After an another 8 h incubation, the culture medium was replaced with 100 μL fresh medium, and the cells were cultured for 48 h for assessment of RNAi. *Renilla* and *Firefly* luciferase gene expressions in the cells were measured by the Dual-Glo Luciferase Assay System (Promega).

### 3.5. Luciferase Activity Assay

To detect the *Firefly* luciferase activity as an intraplasmid control, 50 μL of Dual-Glo Luciferase reagent-1 (beetle luciferin) was added to each well containing 100 μL culture medium in 96-well multiplates. The plates were incubated in the dark for 10 min at room temperature. Luminescence emitted from the *Firefly* luciferase catalytic reaction was measured for 1 s for each well on a microplate reader (Wallac 1420 ARVO MX; Perkin Elmer, Waltham, MA, USA). To measure the *Renilla* luciferase activity and to quench the luminescence from the *Firefly* luciferase catalytic reaction, 50 μL of Dual-Glo Stop and Glo reagent-2 (containing coelenterazine) was added to each well. The luminescence arising from the *Renilla* luciferase catalytic reaction was measured in the same way as described above for *Firefly* luciferase activity, and normalized by the luminescence of *Firefly* luciferase activity in each well. The RNAi efficacy of Peptide-siRNAs toward the *Renilla* luciferase was assessed as a percentage of the control (siRNAs nontreated) sample.

### 3.6. Confocal Microscopy and Flow Cytometry

To prepare FAM-labeled 21-nt and 27-nt siRNAs including peptide conjugates, antisense 21-nt and 27-nt ssRNAs were labeled with 5′-fluorescein phosphoramidite at the 5′-end. The FAM-labeled antisense 21-nt and 27-nt ssRNAs were annealed with peptide-conjugated and nonmodified sense 21-nt and 27-nt ssRNAs, respectively, in annealing buffer.

To deliver the prepared siRNAs intracellularly to the HeLa cells, 200 pmol of each 21-nt or 27-nt siRNAs, including Peptide-siRNAs, labeled with FAM was incubated with 2 μL LF2000 in 100 μL Opti-MEM diluted twice for 30 min at room temperature. Then, 100 μL of each sample was added to 900 μL culture medium of HeLa cells (5 × 10^4^ cells) and incubated for 6 h in the dark under a humidified atmosphere (5% CO_2_, 37 °C). The HeLa cells were washed several times with fresh medium, and the intracellularly incorporated quantity of siRNAs, including Peptide-siRNAs, labeled with FAM in HeLa cells was examined under a fluorescent confocal microscope (IX70; Olympus, Tokyo, Japan). 

Another approach was used to investigate the intracellular delivery of 21-nt and 27-nt siRNAs, including Peptide-siRNAs, labeled with FAM by flow cytometry (FACSAria; BD Biosciences, Franklin Lakes, NJ, USA). The forward- and side-scatter parameters were adjusted to accommodate the inclusion of each of the dissociated cell lines with the aid of FAM as a marker. Five-thousand cells were analyzed and no cells were excluded from the analysis. Data were collected and analyzed using FACSDiva software (BD Bioscience).

## 4. Conclusions

The covalent conjugation of peptide to 27-nt siRNA at the 5′-end of the sense strand can open the way to develop a new generation of modified siRNAs. The modified 27-nt siRNAs appeared to be cleaved to 21-nt siRNAs essential for RNAi; peptides used for modification were out off from the core region of the siRNAs. The 27-nt Peptide-siRNA had the properties of excellent membrane permeability and accumulation in the cytoplasm to promote the RNAi mechanisms owing to accelerated RISC selection through the Dicer cleavage. Although the 21-nt Peptide-siRNA has excellent properties as RNAi molecules, the 27-nt Peptide-siRNA excel in all things, such as recognition by Dicer, cell membrane permeability, and RNAi efficacy, compared with the 21-nt Peptide-siRNA. In particular, the finding of a high level of gene-silencing effect by the 27-nt Peptide-siRNA was notable, since it suggested that the 27-nt Peptide-siRNA might be applicable to various target genes, including endogenous genes. We are certain that the 27-nt Peptide-siRNA can be useful as a new generation of RNAi molecules that overcome some of the limitations of RNAi technology.
